# Fisetin Regulates Gut Microbiota and Exerts Neuroprotective Effect on Mouse Model of Parkinson’s Disease

**DOI:** 10.3389/fnins.2020.549037

**Published:** 2020-12-14

**Authors:** Tian-Jiao Chen, Ya Feng, Te Liu, Ting-Ting Wu, Ya-Jing Chen, Xuan Li, Qing Li, Yun-Cheng Wu

**Affiliations:** ^1^Department of Neurology, Shanghai General Hospital, Shanghai Jiao Tong University School of Medicine, Shanghai, China; ^2^Shanghai Geriatric Institute of Chinese Medicine, Longhua Hospital, Shanghai University of Traditional Chinese Medicine, Shanghai, China; ^3^The State Key Laboratory of Medical Neurobiology, The Institutes of Brain Science and the Collaborative Innovation Center for Brain Science, Shanghai Medical College, Fudan University, Shanghai, China

**Keywords:** Parkinson’s disease, fisetin, gut microbiota, neuroprotective, biomarker

## Abstract

Previous studies have reported the anti-oxidant, anti-inflammatory, and anti-cancer effects of fisetin. However, the therapeutic efficacy of fisetin in Parkinson’s disease (PD) is unclear. In this study, we demonstrated that fisetin could markedly alleviate 1-methyl-4-phenyl-1,2,3,6-tetrahydropyridine (MPTP)-induced dopaminergic neurodegeneration in mice. To confirm the reported correlation between gut microbiota and PD, the bacterial DNA in the fresh feces of mice from each group was subjected to 16S rRNA (V3 and V4 regions) sequencing. The results revealed that fisetin changed the number, diversity, and distribution of gut microbiota in MPTP-induced mice model of PD. The alpha and beta diversity analyses showed that the fisetin intervented MPTP group gut microbiota exhibited a significantly higher abundance of Lachnospiraceae and a significantly lower abundance of uncultured_bacterium_g_Escherichia-Shigella and uncultured_bacterium_g_Bacillus than the MPTP group gut microbiota. These findings indicated that fisetin exerts a neuroprotective effect on neurodegeneration by altering the composition and diversity of gut microbiota. Thus, fisetin could be a potential novel therapeutic for PD.

## Introduction

Parkinson’s disease (PD) is the second most common neurodegenerative disease among the elderly (Kalia and Lang, 2015). In addition to exhibiting motor symptoms, such as bradykinesia, resting tremors, and rigidity, patients with PD exhibit non-motor symptoms, such as gastrointestinal dysfunction, sleep disorders, autonomic dysfunction, and sensory disturbances (Schapira et al., 2017). The main pathological feature of PD is the accumulation of alpha-synuclein, which results in the degeneration of dopaminergic neurons in the substantia nigra (SN) (Michel et al., 2016). However, the molecular mechanism underlying PD has not been fully elucidated and there are no known effectively neuroprotective therapies for PD (Kalia and Lang, 2015).

Oxidative stress, mitochondrial dysfunction, autophagy impairment, and inflammation are reported to be involved in the pathophysiological progression of neuronal degeneration in PD. However, the detailed mechanism underlying neurodegeneration in PD is still not fully elucidated. Based on the premotor symptoms of PD, such as gastrointestinal (GI) disorders, it was hypothesized that the site of disease origin may be outside the central nervous system (CNS), such as the gastrointestinal tract (Braak et al., 2006). Recent evidence suggests a correlation between gut microbiota and CNS disorders. The microbiota-gut-brain axis plays an important role in maintaining homeostasis (Fung et al., 2017). Previous studies have reported that gut microbiota can help to maintain the integrity of the blood-brain barrier (BBB), regulate the expression of brain-derived neurotrophic factor (BDNF) (Braniste et al., 2014), and affect the growth and function of immune cells in CNS (Erny et al., 2015). Recent studies have also reported that gut microbiota imbalance is related to many neurodegenerative diseases, such as PD, Alzheimer’s disease (AD), and multiple sclerosis (MS) (Cryan et al., 2019). The patients with PD and healthy control subjects exhibit differential gut microbiota composition. Moreover, the differentially abundant taxa are reported to markedly vary between patients with PD and healthy control subjects. Compared to the healthy control subjects, patients with PD exhibit alterations in the gut microbial community, including a decreased abundance of the Prevotellaceae family, and an increased abundance of *Akkermansia* genus, Verrucomicrobiaceae family, *Bifidobacterium* genus and Lactobacillaceae family (Aho et al., 2019). The relative abundance of Enterobacteriaceae family in PD patients is reported to be positively correlated with the severity of postural instability and gait difficulty (Scheperjans et al., 2015). Sun et al. (2018) revealed fecal microbiota transplantation (FMT) can protect PD mice by inhibiting neuroinflammation. Another study also demonstrated that germ-free mice can develop PD symptoms by receiving FMT from PD patients (Sampson et al., 2016). This suggests that gut microbiota imbalance is involved in PD pathogenesis and affect brain function. Thus, drugs or compounds targeted at modulating the gut microbiota could be a potential therapeutic strategy for PD.

Fisetin, a bioactive flavonoid, is abundant in various vegetables and fruits (Adhami et al., 2012). Fisetin is an effective metal chelating agent, free radical scavenger, and enzyme inhibitor. Additionally, fisetin is used for treating various chronic diseases, such as cardiovascular disease and diabetes. Recent studies have demonstrated the neurotrophic activity of fisetin. Additionally, fisetin is reported to promote the differentiation of PC12 cells by activating the Ras-ERK pathway (Ahmad et al., 2017). It has been reported that flavonoids. inhibits Aβ_25–35_-induced neuronal death by changing the electrophysiological characteristics of potassium channels and voltage-gated sodium (Wang et al., 2019). Furthermore, fisetin can alleviate rotenone-induced behavioral deficits, mitochondrial dysfunctions, and aberrant dopamine levels in the rat model of PD (Alikatte et al., 2020). Fisetin is also reported to increase the dopamine level in the striatum of 1-methyl-4-phenyl-1,2,3,6,-tetrahydropyridine (MPTP) mouse model of PD (Maher, 2017). However, the mechanism underlying the neuroprotective effect of fisetin on dopaminergic neurons has not been elucidated.

In this study, we used the MPTP mouse model of PD to investigate the neuroprotective effects of fisetin and examined the potential mechanisms associated with the gut microbiota imbalance.

## Materials and Methods

### Modeling PD in Mice

Twelve-week-old male C57BL/6 mice were purchased from Modelorg (Shanghai Model Organisms Company, Shanghai, China). The mice were randomly divided into three groups with each group comprising eight mice as follows: F group (fisetin + MPTP), orally administered with fisetin (100 ng/kg bodyweight) (Sigma-Aldrich, St Louis, United States) for 30 consecutive days; MPTP group (PBS + MPTP), orally administered with phosphate-buffered saline (PBS PH7.4) for 30 consecutive days; normal control group, simultaneous intragastric and intraperitoneal administration of PBS. On day 25 post-PBS/fisetin administration, the F and MPTP groups were intraperitoneally injected with MPTP (30 mg/kg bodyweight) (Sigma-Aldrich, St Louis, United States) once a day for five consecutive days. During administration of MPTP, mice of the fisetin + MPTP group were also given fisetin. All traumatic operations were performed under anesthesia after intraperitoneal injection of pentobarbital sodium (50 mg/kg bodyweight). Every effort was made to minimize the suffering of the animals. This study was approved by the Ethics Committee of Shanghai Model Organisms Company (IACUC No.2018-0005). All experiments were performed in compliance with China’s National Science and Technology Commission Laboratory Animal Regulations.

### Behavioral Assessments

#### Open Field Test

The behavioral experiments were performed on day 1 after the last MPTP administration. The open field test is a useful method to assess spontaneous locomotor activity (Asakawa et al., 2016). Before the experiment, the mice were allowed to adapt to the experimental environment for half an hour. The mice were placed in an experimental box (40 cm × 40 cm) and their movement was recorded using the open field working station (MED Associates, Georgia, VT, United States). The total distance and mean velocity of the movement were analyzed over a period of 15 min.

#### Pole Test

The pole test is often used to measure bradykinesia in mouse model of PD (Ogawa et al., 1985). Before MPTP injection, the mice are trained to climb the pole with their head pointing toward the top of the pole (height 100 cm with a diameter of 1 cm). The mice are then trained to turn around and climb down to the bottom of the pole three times. On day 1 after the last administration of MPTP, the time taken by the mice to turn around and the total time taken to climb from the top to the bottom of the pole were recorded. Each mouse was tested three times at 1 h intervals.

#### Hanging Wire Test

The hanging wire test is used to evaluate the coordination ability of mice. The front paw of the mouse was placed at the center of the horizontal wire (2 mm in diameter, 50 cm in length, 35 cm from the bottom). The mice tend to support themselves using their hind claws to avoid falling and walk along the wire to the platform. The number of times the mice drops from the wire (up to 10 times) and the number of arrivals (up to 10 times) in 180 s were recorded. The total score for falls and arrivals was obtained using the following formula: (10 falls + arrivals) (Klein et al., 2012).

### Nissl Staining

Brain samples were collected and fixed overnight in 4% paraformaldehyde at −4°C for the preparation of paraffin-embedded sections. The paraffin-embedded sections were dewaxed, hydrated, stained with 0.1% Cresyl violet solution buffer for 10 min, soaked in absolute ethanol for 5 min, and cleared in xylene for 5 min. The paraffin sections were sealed with neutral gum and observed under a light microscope (BX51, Olympus, Tokyo, Japan).

### Protein Extraction and Western Blotting Analysis

The mice were sacrificed on day 1 after the last administration of MPTP. The brain tissues of the striatum were isolated and stored at −80°C. The brain tissues were homogenized in 1X RIPA lysis buffer containing 1 mM phenylmethylsulfonyl fluoride (PMSF) and phosphatase inhibitor cocktail (Roche, Basel, Switzerland). The lysate was centrifuged at 4°C and 12,000 *g* for 15 min. Equal amounts of protein from all groups were subjected to sodium dodecyl sulfate-polyacrylamide gel electrophoresis (SDS-PAGE) using 12% gel. The resolved proteins were transferred to a 0.45-μm polyvinylidene fluoride (PVDF) membrane. The membrane was blocked using 5% (w/v) skimmed milk to inhibit non-specific binding. Next, the membrane was incubated overnight at 4°C with the following primary antibodies: mouse anti-tyrosine hydroxylase (TH) (Sigma, 1:000) and mouse anti-β-actin (Proteintech, 1:1000) antibodies. The membrane was then incubated with the HRP-conjugated goat anti-mouse (CST, United States 1:1000) secondary antibodies for 1 h. The proteins were visualized using the enhanced chemiluminescence (ECL) test kit and Invitrogen iBright 1,500 system.

### Gut Microbiota Analysis

The fresh fecal samples of all mice were collected on days 3 and 5 post-MPTP administration and stored at −80°C. The fecal bacterial composition was determined by amplifying and sequencing the V3 and V4 regions of the bacterial 16S rRNA. The sequence of the primers used for polymerase chain reaction (PCR) amplification is as follows: F primer, 5′-ACTCCTACGGGAGGCAGCA-3′; R primer, 5′-GGACTACHVGGGTWTCTAAT-3′. The Illumina HiSeq platform was used for high-throughput sequencing of bacterial 16S rRNA. The valid sequence data were classified as operational taxonomic units (OTUs) at 97% similarity level using the UCLUST (Edgar, 2010) software. Based on the results of OTUs, the genetic relationship among bacterial species and the differences among species were analyzed to obtain the relationship between bacterial classification, bacterial relative abundance, and bacterial community. The α diversity index of the samples was evaluated using the Mothur software (version, v.1.30). The α diversity index indicates the species richness and diversity within a sample. To compare the diversity index among samples, the number of sequences contained in each sample was standardized. The indicators, including rarefaction, OTU rank, and Shannon curves, and the ACE, Shannon, Chao1, and Simpson indices were calculated. The QIIME (Caporaso et al., 2010) software was used for beta diversity analyses, including unweighted pair-group method with arithmetic mean (UPGMA), NMDS (Looft et al., 2012), PCoA (Sakaki et al., 1994), PCA, and heatmaps of redundancy analysis (RDA)-identified key OTUs, to compare the similarity of species diversity of different samples. The significant biomarkers were determined by line discriminant analysis (LDA) effective size (LEfSe). The biomarkers and rich flora were determined using the LDA threshold >4.

### Statistical Analysis

The data are expressed as mean ± standard error (SEM). The data across groups were compared by one-way analysis of variance (ANOVA), followed by the least significant difference (LSD) multiple comparison test. All statistical analyses were performed in Prism 6.0 (GraphPad Software, Inc., San Diego, CA, United States). The difference was considered statistically significant when the *p*-value was less than 0.05.

## Results

### Fisetin Alleviates MPTP-Induced Behavioral Impairments in Mice

**FIGURE 1:**
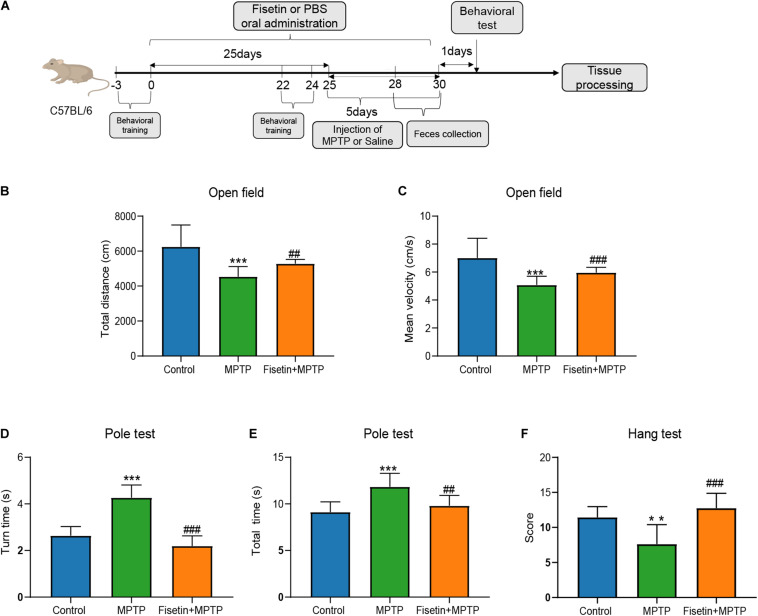
Fisetin attenuates 1-methyl-4-phenyl-1,2,3,6,-tetrahydropyridine (MPTP)-induced behavioral impairments in mice. **(A)** Experimental procedure time line; **(B)** The total distance, and **(C)** the mean velocity of mouse movement in the open field test; **(D)** The total time taken to climb the pole, and **(E)** the turn time in the pole test; **(F)** The score of wire hanging test. Data are expressed as mean ± standard error of mean (SEM). ^∗∗^*p* < 0.01 and ^∗∗∗^*p* < 0.001 compared with the control group; ##*p* < 0.01 and ###*p* < 0.001 compared with the MPTP group (*n* = 8).

The mice were intraperitoneally injected with MPTP to establish the PD model. And changes of body weight between groups at different time point was presented as Supplementary Data 1. The effect of fisetin on MPTP-induced behavioral impairments was assessed using the open field, pole, and hanging wire tests. The open field test is often used to evaluate the spontaneous behavior of mice, which indicates the inactivity of mice. The total distance and mean velocity of movement of the MPTP group were significantly lower than those of the control group. The administration of fisetin significantly attenuated the MPTP-induced locomotor activity impairments in mice (Figures 1B,C). Further, we evaluated the effect of fisetin on MPTP-induced bradykinesia and coordination deficits in mice by the pole and hanging wire tests, respectively. The administration of MPTP prolonged the turn time and total time spent on the pole, which was attenuated by fisetin treatment (Figures 1D,E). The score of MPTP group in the hanging wire test was lower than that of the control group. The administration of fisetin attenuated the MPTP-induced coordination impairments in mice (Figure 1E). The results of behavioral experiments revealed that fisetin treatment can improve the MPTP-induced motor behavior impairments in mice.

### Fisetin Attenuates MPTP-Induced Dopaminergic Neurodegeneration in SN and Striatum

**FIGURE 2:**
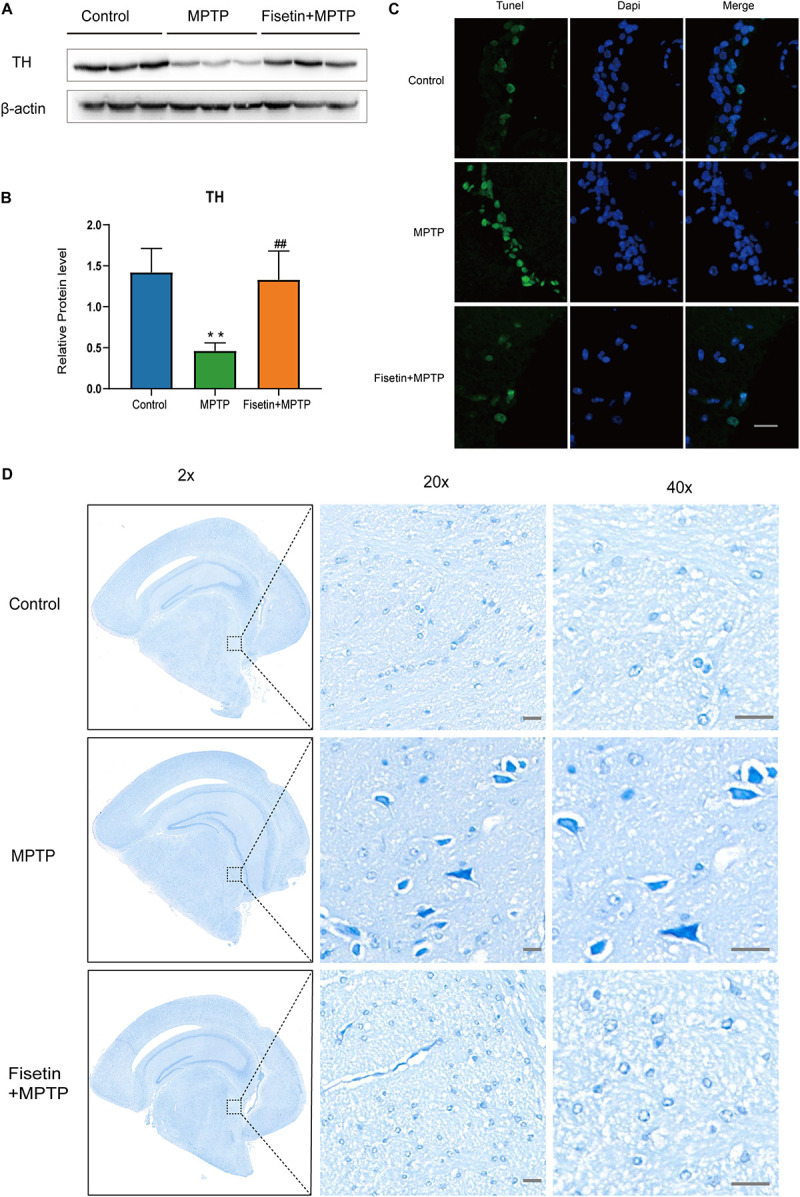
Fisetin attenuates 1-methyl-4-phenyl-1,2,3,6,-tetrahydropyridine (MPTP)-induced dopaminergic neurodegeneration **(A,B)** The expression levels of tyrosine hydroxylase (TH) in the striatum; **(C)** TUNEL staining to assess apoptosis. Scale bar = 100 μm; **(D)** Nissl staining to assess neuronal injury. Scale bar = 20 μm.

The protein level of TH was estimated by western blotting. Treatment with MPTP significantly decreased the TH levels in mice. The F group exhibited improved TH levels when compared to the MPTP group (Figures 2A,B). The dopaminergic neurodegeneration was assessed by TUNEL staining, Nissl staining and immunofluorescence staining. As shown in Figure 2C, treatment with MPTP increased the number of TUNEL-positive (apoptotic) neurons. Contrastingly, the fisetin group exhibited a significantly lower number of TUNEL-positive neurons than the MPTP group. Nissl staining is used to stain the Nissl body located in the cytoplasm of neurons. The MPTP group exhibited a significantly higher amount of Nissl-stained dark neurons than the control group. The administration of fisetin attenuated the MPTP-induced increase in the number of Nissl-stained dark neurons (Figure 2D). Next, we used immunofluorescence staining to detect TH positive neurons in SN of mice. We observed the decrease of dopaminergic neurons in MPTP group, while the number of TH positive neurons increased in fisetin and MPTP group (Supplementary Figure 2). These results indicated that fisetin can alleviate MPTP-induced dopaminergic neurodegeneration in the SN-striatum axis of PD mice.

### Fisetin Affects the Number and Composition of Gut Microbiota in MPTP-Treated Mice

**FIGURE 3:**
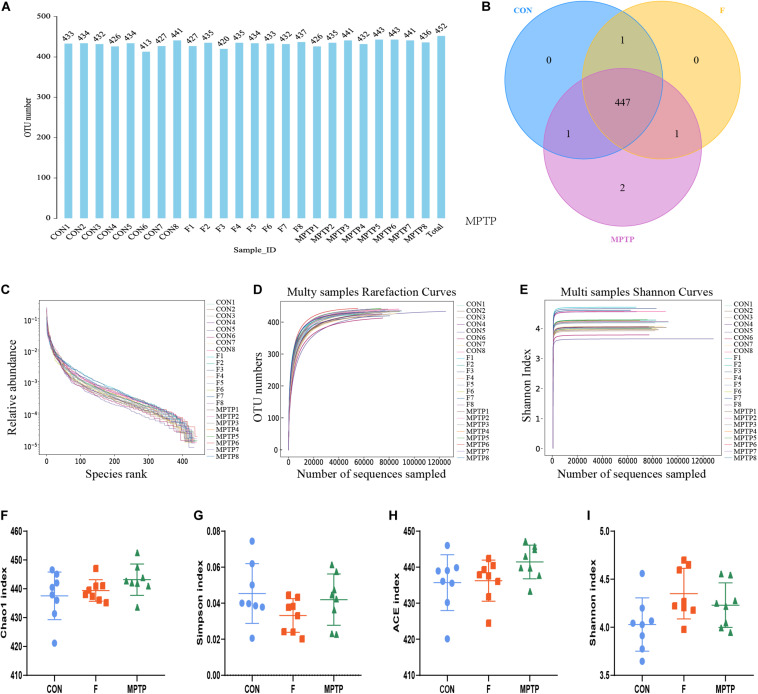
The alpha diversity analysis of gut microbiota isolated from the feces of mice of each group via 16S rRNA (V3 and V4 regions) sequencing. **(A)** Operation taxonomic unit (OTU) statistic results; **(B)** OTU-Venn comparison results; **(C)** OTU rank abundance curve statistic results; **(D)** OTU rarefaction curve statistics; **(E)** OTU Shannon index statistics; The alpha diversity statistics of OTUs based on Shannon index **(F)**, Chao1 index **(G)**, Simpson index **(H)**, and ACE index **(I)**.

The feces of mice from normal mice (CON), MPTP-treated mice from the PBS intervention group (MPTP), and MPTP-treated mice from the fisetin intervention group (F) were collected. The composition and specific distribution of gut microbiota were evaluated by sequencing the bacterial V3 and V4 regions of the 16S rRNA. In total, 3,127,202 pairs of reads were obtained by sequencing 24 samples. Of these, 2,793,501 clean tags were generated after splicing the paired-end reads and filtering. An average of 116,396 clean tags was obtained with at least 86,445 clean tags obtained from each sample. The sequences were clustered into OTUs with 97% sequence similarity using the QIIME (version 1.8.0) UCLUST software. We generated the OTU rank table, Shannon index curves, rarefaction curves, Simpson curves, Chao1 curves, and ACE curves, the results showed that there were no significant differences in the number of OTUs among the three groups, and the sequencing data of each sample was enough to reflect the species diversity (Figures 3A,C–E). Chao1 and Ace index were used to measure species richness, and Shannon and Simpson index to measure species diversity. As shown in the Figures 3F–I, there were no significant differences in chao1 index, Simpson index, Ace index and Shannon index of α-diversity among groups. The results showed that there was no significant difference in α-diversity among the three groups. However, the Venn diagram showed that there were no significant differences in the number of OTUs between the control and F groups. The number of OTUs in the MPTP group was two more than that in the control and F groups (Figure 3B). The two bacterium are Kozakia_baliensis belonging to the family Acetobacteraceae and uncultured_bacterium_g_Bacillus belonging to the family Bacillaceae. The Venn diagram revealed that one bacterium (uncultured_bacterium_g_[Eubacterium]_ruminantium_group belonging to the family Lachnospiraceae) was detected in the F and control groups, which was not detected in the MPTP group. The administration of fisetin affected the abundance and composition of gut microbiota in MPTP-treated mice.

### Fisetin Alters the Distribution and Diversity of Gut Microbiota in MPTP-Treated Mice

**FIGURE 4:**
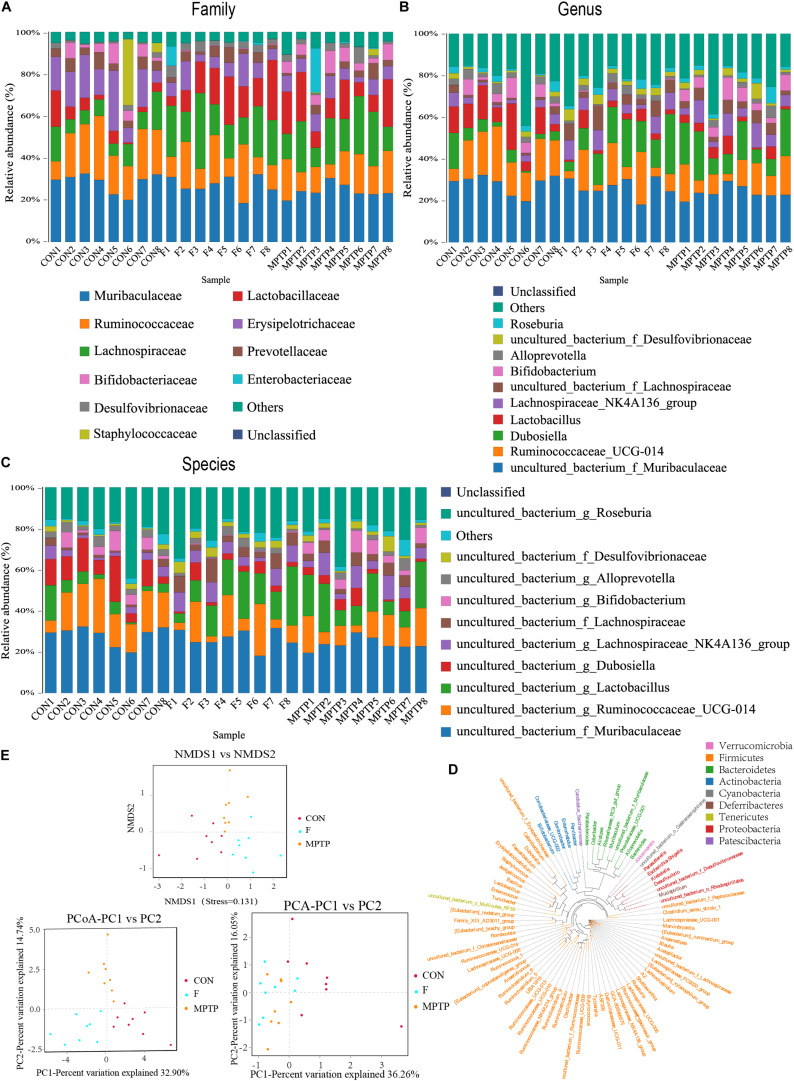
Fisetin alters the distribution and diversity of gut microbiota in MPTP mice. Community clustering analysis of operational taxonomic units (OTUs) at the family **(A)**, genus **(B)**, and species **(C)** levels; **(D)** Phylogenetic analysis of OTUs; **(E)** Beta diversity analysis of OTUs based on Unweighted UniFrac.

**FIGURE 5:**
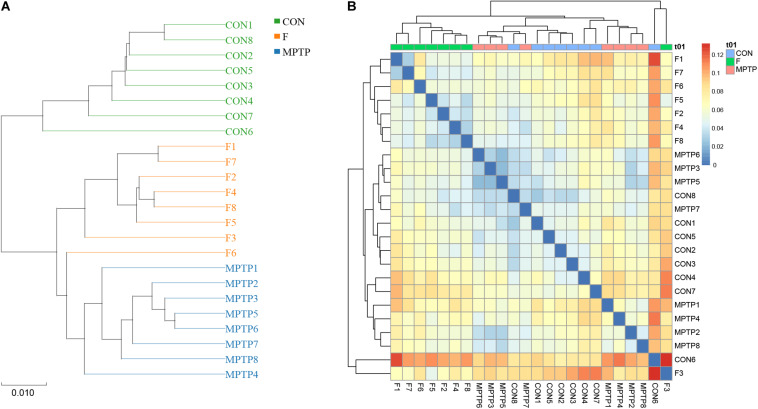
Hierarchical clustering analysis of the 16S rRNA (V3 and V4 regions) sequencing results. **(A)** Unweighted pair-group method with arithmetic mean (UPGMA) analysis. **(B)** Heatmap analysis of samples from various groups based on the distance algorithm (unweighted).

Each OTU can be classified into a species by comparing the microbial reference sequence data and representative sequences of OTUs. The community composition of each sample was determined. The species abundance tables at different classification levels (phylum, class, order, family, genus, and species) were generated using the QIIME software. The community structure of samples at different taxonomic levels was plotted using the R software. Compared to the MPTP group, the F group exhibited a significantly higher relative abundance of Lachnospiraceae family and a significantly lower relative abundance of Bifidobacteriaceae, Enterobacteriaceae and Bacillaceae families (Figure 4A). The differential microbiota was further analyzed at the genus and species levels. At the genus level, the F group exhibited a higher relative abundance of uncultured_bacterium_f_Lachnospiraceae, [Eubacterium]_ruminantium_group, and Marvinbryantia, while a significantly lower relative abundance of Bifidobacterium, Escherichia_Shigella and Bacillus than the MPTP group (Figure 4B). Analysis at the species level showed that compared with Group MPTP, the relative abundance of uncultured_bacterium_f_Lachnospiraceae, uncultured_ bacterium_g_Marvinbryantia, uncultured_bacterium_g_ [Eubacterium]_ruminantium_group (all of them belonging to the family Lachnospiraceae) was significantly increased in the guts of individuals in Group F, while the relative abundance of uncultured_bacterium_g_Bifidobacterium (family Bifidobacteriaceae), uncultured_bacterium_g_Escherichia_ Shigella (family Enterobacteriaceae) and uncultured_bacterium_ g_Bacillus (family Bacillaceae) were significantly decreased (Figures 4C,D and Supplementary Table 1). The Binary-Jaccard algorithm was used to analyze the differences in the microbial communities among the three groups. The analyses mainly include non-metric multi-dimensional scaling (NMDS), principal component analysis (PCA), and principal coordinate analysis (PCoA). The results of these analyses revealed that there were significant differences in the distribution of microbial communities among the three groups (Figure 4E). The hierarchical UPGMA cluster analysis revealed that the gut microbiota of the Groups F and CON had high homology and closer genetic relationship (Figures 5A,B).

**FIGURE 6:**
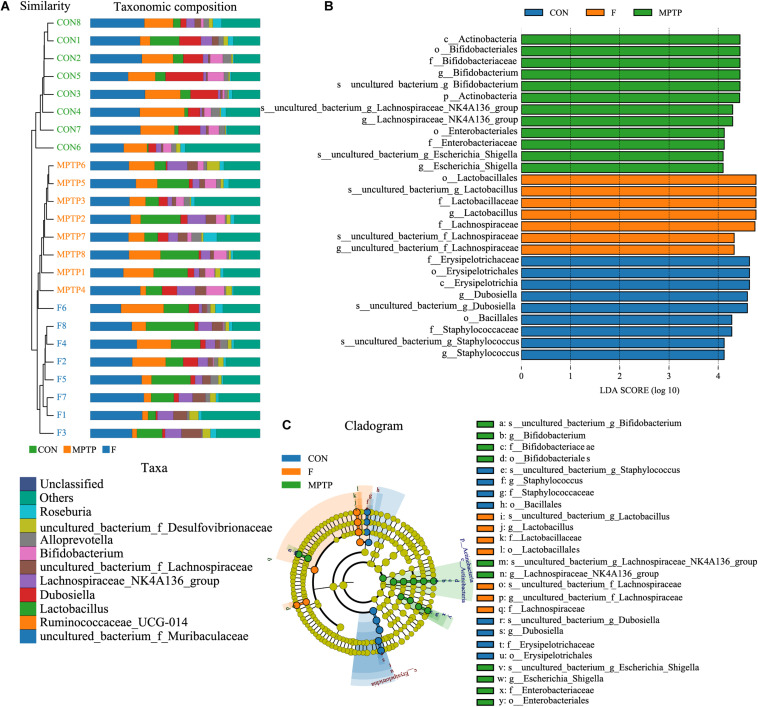
The analysis of differences in gut microbiota from feces of mice from each group. **(A)** Combined unweighted pair-group method with arithmetic mean (UPGMA) cluster tree and histogram analysis; **(B)** Line discriminant analysis (LDA) effect size (LEfSe) analysis of samples from various groups; **(C)** Phylogenetic branches from the LEfSe analysis.

The samples of each group can be clustered hierarchically in Python language to reveal the characteristics of the microbial population of each group using the combination of UPGMA cluster tree and histogram plots. The integrated analysis revealed that uncultured_bacterium_f_Lachnospiraceae was the dominant bacteria in F group, while the abundance of Bifidobacterium was relatively lower than the MPTP group (Figure 6A). These results are consistent with those of the species abundance table generated by the QIIME software. Line Discriminant Analysis (LDA) Effect Size (LEfSe) was used to find high-dimensional significant biomarkers among different groups. The LDA score is used to represent the influence of different species. The LDA scores greater than 4 are considered important biomarkers. The LDA score distribution and Cladogram analysis revealed that the fisetin group gut microbiota had a significantly higher abundance of Lachnospiraceae family and a significantly lower abundance of Bifidobacterium and Escherichia_Shigella than the MPTP group gut microbiota (Figures 6B,C). The distribution of the abovementioned microbiota was opposite in the MPTP group.

### Fisetin Can Alter the Expression of 16S Functional Genes and Metabolic Signaling Pathways in Intestinal Microbes of PD Mice

**FIGURE 7:**
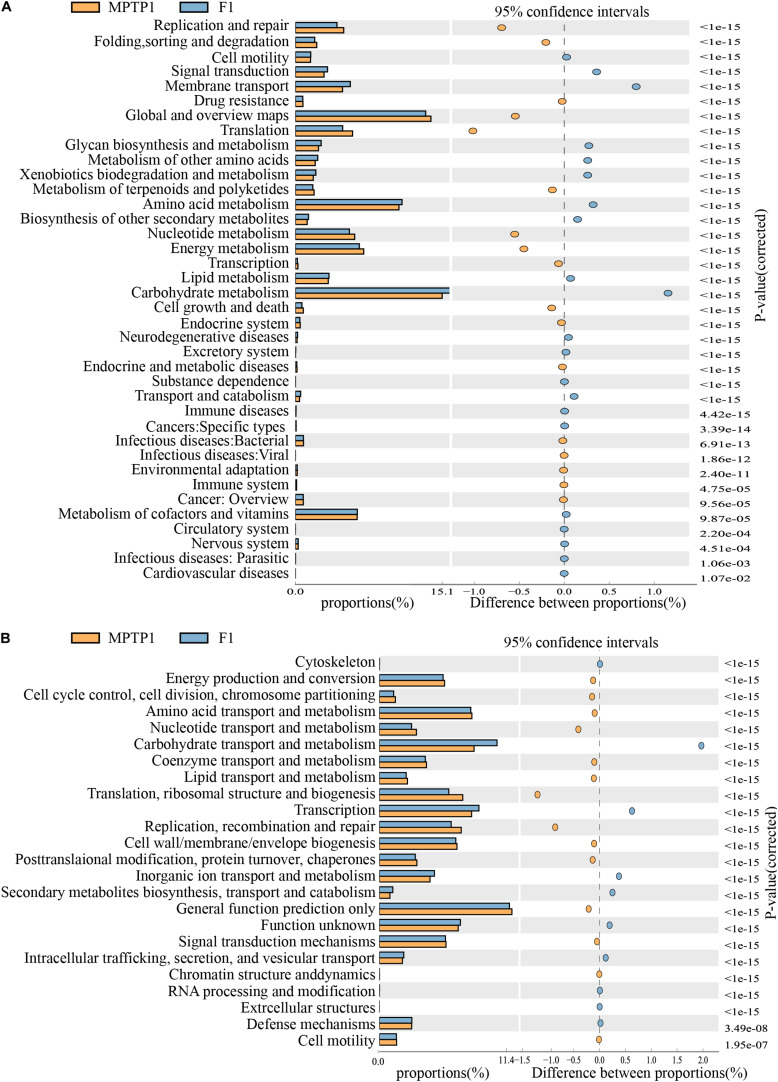
Fisetin can alter the expression of 16S functional genes and metabolic signaling pathways in intestinal microbes of Parkinson’s disease (PD) mouse model. **(A)** KEGG functional prediction analysis. **(B)** Clusters of orthologous groups (COGs) of proteins functional prediction analysis.

The KEGG metabolic pathway analysis revealed differences in the effect of microbes on the metabolic pathways among the three groups. The analysis revealed that the F group gut microbiota can upregulate metabolic pathways, such as carbohydrate metabolism and environmental information processing (such as membrane transport) and downregulate translation, replication, and repair of genetic information (Figure 7A and Supplementary Table 2) when compared to the MPTP group gut microbiota.

The analysis of clusters of orthologous groups (COG) of proteins enables the identification of distribution and abundance of homologous protein clusters in the microbiota (Figure 7B and Supplementary Table 3). The abundance of proteins associated with carbohydrate transport and metabolism in the F group gut microbiota was significantly higher than that in the MPTP group gut microbiota. However, the abundance of proteins associated with replication, recombination and repair, translation, ribosomal structure, and biogenesis in the fisetin group gut microbiota was significantly lower than that in the MPTP group gut microbiota.

## Discussion

Several studies have reported the vital role of non-motor symptoms, including hyposmia, gastrointestinal dysfunction, and constipation, which precede the onset of motor symptoms, in PD (Klingelhoefer and Reichmann, 2017; Pereira et al., 2017). Among the non-motor symptoms, gastric motility disorders, especially constipation, are reported in 70–100% of patients with PD (Fasano et al., 2015; Li et al., 2017). Additionally, some studies have confirmed the presence of Lewy bodies in the enteric nervous system (Keshavarzian et al., 2015). Liao et al. had shown that oral Lactobacillus plantarum PS128 attenuates oxidative stress and neuroinflammation in the nigra-striatum pathway induced by MPTP and inhibits the increase of Enterobacteriaceae and lipopolysaccharide and peptidoglycan biosynthesis-related microbial modules induced by MPTP (Liao et al., 2020). Pu et al. (2019) confirmed that antibiotic-induced microbiome depletion can significantly improve the decrease of dopamine transporter (DAT) immunoreactivity in the striatum and TH immunoreactivity in the SN. These findings indicate the involvement of the microbiota-gut-brain axis (MGBA) in the pathophysiology of PD, which can be a potential therapeutic target for PD. Fisetin, a flavanol from fruits and vegetables, exerts anti-oxidant, anti-inflammatory, and anti-aging effects in MPTP induced mice model of PD (Mukhtar et al., 2015; Si et al., 2019). However, the underlying mechanism is unclear. In this study, the effects of orally administered fisetin on MPTP-induced PD were examined in mice and explored its association with gut microbiota. Previous study demonstrated the neuroprotective effect of fisetin in the MPTP model of PD, which may be closely related to attenuation of α-synuclein expression. The mechanisms study showed the potential inhibition of apoptotic and inflammatory pathways (Patel et al., 2012). Consistent with previous study, in our experiment, the behavioral test results suggested that fisetin could attenuate MPTP-induced behavior impairments in mice. The results of western blotting, Nissl staining, and TUNEL staining also confirmed the role of fisetin on MPTP-induced dopaminergic apoptosis. Next, the bacterial DNA in the fresh feces collected from the three groups were subjected to 16S rRNA (V3 and V4 regions) sequencing to assess the composition of gut microbiota and the differences in the distribution of specific flora. In addition to affecting the diversity and distribution of microbiota, fisetin treatment affected the abundance and composition of gut microbiota in the MPTP mouse model of PD. The alpha and beta diversity analyses revealed that the F group gut microbiota exhibited a significantly higher abundance of Lachnospiraceae and a significantly lower abundance of uncultured_bacterium_g_Bacillus and uncultured_bacterium_g_Escherichia-Shigella than the MPTP group gut microbiota. Consistent with these results, a recent study also reported that patients with PD exhibited a lower abundance of Lachnospiraceae than healthy control (Hill-Burns et al., 2017). Additionally, Lin et al. have demonstrated that compared with healthy control, the abundance of Lachnospiraceae was reduced by 42.9%, while that of Bifidobacteriaceae was enriched in patients with PD (Lin et al., 2018). Therefore, we hypothesized that there is a strong correlation between Lachnospiraceae and pathogenesis of PD and that targeting this microbe could be a new therapeutic strategy for PD.

Lachnospiraceae is a beneficial butyrate-producing bacterium that is associated with gut health. Butyrate is an energy source for the gut epithelium, which inhibits NF-KB activation to reduce gut inflammation (Lin et al., 2018). Srivastav et al. (2019) had shown that the application of probiotics can significantly increase the metabolite BHB of butyrate and confirmed that butyrate can protect against MPTP neurotoxicity by preventing dopaminergic neuronal loss and dopamine depletion, reducing gliosis proliferation in SN and up-regulating neurotrophic factors. The results of this study indicated that fisetin may exert its neuroprotective effect by increasing the abundance of Lachnospiraceae, which alleviates gut inflammation and reduces the production of toxic substances. This allows the spread of Lachnospiraceae from the gut to the brain. Moreover, several studies have reported that the depletion of short-chain fatty acids (SCFA) contributes to the pathogenesis of PD because it could potentially induce inflammation and microglial activation, which results in gastrointestinal disorders, such as leaky gut and constipation (Barcenilla et al., 2000; Duncan et al., 2002; Unger et al., 2016; Lin et al., 2018). Interestingly, SCFA is produced by gut bacteria (mainly Lachnospiraceae) that mainly metabolize carbohydrates. This is consistent with the results of Hill-Burns who reported that the abundance of intestinal Lachnospiraceae in patients with PD decreased with a concomitant decrease in the SCFA levels (Hill-Burns et al., 2017). Several studies have reported the vital role of SCFA in the development of PD. Thus, replenishing the microbiome with SCFA-producing bacteria could be a potential preventive strategy for PD. This was consistent with the results of this study, which demonstrated that the F group gut microbiota exhibited a higher abundance of Lachnospiraceae and higher carbohydrate metabolism than the MPTP group. The increased abundance of Lachnospiraceae may produce enhanced SCFA levels and exert neuroprotective effects. Additionally, Lachnospiraceae can promote the aggregation of regulatory T cells in the colon and reduce the immunoglobulin E level (Wu et al., 2013; Stiemsma et al., 2016), while regulatory T cells can delay the dopaminergic neurodegeneration in PD (Christiansen et al., 2016).

In addition to the association of the Lachnospiraceae family with PD development, some studies have reported the negative correlation between the abundance of Lachnospiraceae and PD duration (Scheperjans et al., 2015; Hill-Burns et al., 2017). Li et al. reported that patients with PD exhibited a significantly high abundance of the intestinal conditional pathogens, such as Enterococcus, Escherichia-Shigella, and Proteus. Additionally, the abundance of these pathogens was positively correlated with PD duration and unified Parkinson’s disease rating scale (UPDRS) score (Li et al., 2017). Gut microbiota can produce and secrete extracellular amyloid proteins, which can not only promote bacterial colonization, adhesion and biofilm formation, but also promote tissue invasion, infectivity, and induce misfolding of aggregation-prone proteins in the host (Taylor and Matthews, 2015; Chen et al., 2016). Bacillus and Escherichia-Shigella secrete amyloid protein and promote the occurrence of diseases. Amyloid proteins can induce oxidative stress, activate microglia, and release inflammatory factors such as TNF-α, IL-1 and IL-6 to increase to increase permeability of intestinal epithelial and BBB (Harach et al., 2017; Van Gerven et al., 2018). Escherichia-Shigella can cause diarrhea and produce Shiga toxin, which can cause functional damage to the CNS of rabbits and rodents (Lee and Tesh, 2019). It is suggested that Shigella, a conditional pathogen, can produce endotoxin, promote intestinal inflammation, and damage the CNS in patients with PD. A recent study indicated that colonization of curli-producing Escherichia coli accelerates aSyn pathology in the gut and brain. Escherichia coli needs Curli expression to exacerbate α-Syn-induced intestinal and motor disorders (Sampson et al., 2020). Moreover, epigallocatechin gallate, a plant-derived dietary polypheno can prevent pathology and motor symptoms in Thy1-SNCA mice by blocking amyloidogenic subunit of curli fibers (CsgA) amyloidogenesis and repressing CsgA transcript expression in Escherichia coli (Sampson et al., 2020). This is consistent with the results of this study, which reported that the fisetin group gut microbiota exhibited a lower abundance of Escherichia-Shigella, Bacillus and proteins associated with genetic information processing than the MPTP fisetin group. This indicated that fisetin may inhibit inflammation and alleviate dopaminergic neurodegeneration by inhibiting the growth of harmful microorganisms.

What’s more, several studies have confirmed the antioxidant activity of fisetin and its ability to maintain GSH level under stress through inducing the expression of Nrf2 and ATF4 (Maher, 2017). Whether fisetin can still exert the mentioned effect in PD model still need further studies.

In summary, this study demonstrated that fisetin can protect MPTP-induced dopaminergic neurodegeneration. The potential mechanism underlying the neuroprotective effect of fisetin involves the regulation of the distribution and abundance of Lachnospiraceae, uncultured_bacterium_g_Escherichia-Shigella and uncultured_bacterium_g_Bacillus in the MPTP-induced mouse PD model. And in our further experiments, we would like to explore other possible mechanisms of fisetin for PD, such as antioxidant and anti-inflammatory effect.

## Data Availability Statement

The datasets presented in this study can be found in online repositories. The names of the repository/repositories and accession number(s) can be found in the article/Supplementary Material.

## Ethics Statement

The animal study was reviewed and approved by the Institutional Animal Care and Use Committees (IACUC) of the Shanghai Model Organisms Company (IACUC No. 2018-0005).

## Author Contributions

Y-CW and TL designed the study and revised the manuscript. T-JC conducted the experiments. T-JC and YF performed the data analysis and prepared the manuscript. T-TW and XL assisted with 16S rRNA (V3 and V4 regions) sequencing. XL and T-TW provided the experimental materials and assisted in troubleshooting. All authors have approved the manuscript and declared to take full responsibility for the data generated in this study.

## Conflict of Interest

The authors declare that the research was conducted in the absence of any commercial or financial relationships that could be construed as a potential conflict of interest.
