# Supplementary material for: Fisetin Regulates Gut Microbiota and Exerts Neuroprotective Effect on Mouse Model of Parkinson’s Disease
Source: Front Neurosci. 2020 Dec 14;14:549037. doi: 10.3389/fnins.2020.549037 (PMC7768012; doi:10.3389/fnins.2020.549037)
Supplement: Supplementary file 1 [file Data_Sheet_1.docx]

Supplementary data:


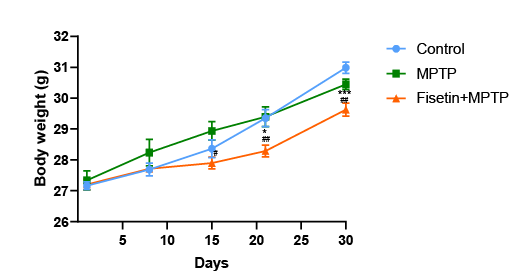


**Figure S1 Changes of body between groups at different time points.**


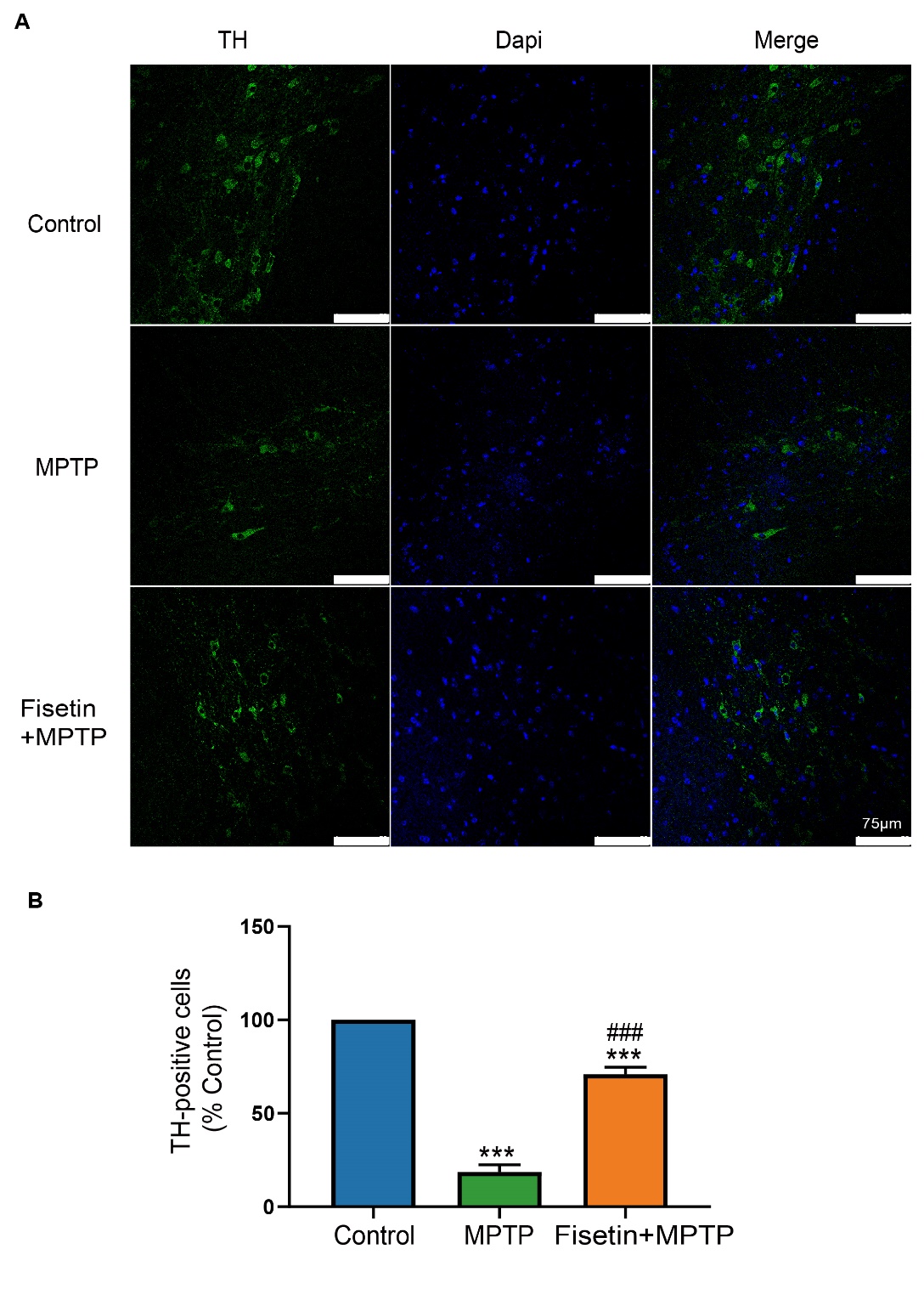


**Figure S2 Fisetin ameliorated MPTP induced dopaminergic injury.** (A) immunofluorescence staining of TH (green) and Dapi (Blue) in the substantia nigra (scale bar: 75 µm); (B) Relative immunoreactivity intensity of TH (n = 3). All data are presented as means ± SEM. ∗p < 0.05 and ∗∗∗p < 0.001, compared with the Control group; ##p < 0.01, compared with the MPTP group.
