# Supplementary material for: Fisetin Regulates Gut Microbiota and Exerts Neuroprotective Effect on Mouse Model of Parkinson’s Disease
Source: Front Neurosci. 2020 Dec 14;14:549037. doi: 10.3389/fnins.2020.549037 (PMC7768012; doi:10.3389/fnins.2020.549037)
Supplement: Supplementary file 2 [file Table_1.docx]

Supplementary Material

## Supplementary table S1: Differential species ( F vs MPTP)

| species | Mean(F) | Variance(F) | Std.err(F) | Mean(MPTP) | Variance(MPTP) | Std.err(MPTP) | P value | Q value |
| --- | --- | --- | --- | --- | --- | --- | --- | --- |
| Kozakia_baliensis | 0.00E+00 | 0.00E+00 | 0.00E+00 | 3.00E-04 | 1.60E-08 | 4.47E-05 | 9.99E-04 | 1.63E-02 |
| uncultured_bacterium_g_Bacillus | 0.00E+00 | 0.00E+00 | 0.00E+00 | 1.04E-03 | 7.48E-08 | 9.67E-05 | 9.99E-04 | 1.63E-02 |
| uncultured_bacterium_g_Bifidobacterium | 2.99E-03 | 3.35E-06 | 6.48E-04 | 5.84E-02 | 6.69E-04 | 9.14E-03 | 9.99E-04 | 1.63E-02 |
| uncultured_bacterium_g_Catenibacterium | 2.43E-05 | 3.42E-10 | 6.54E-06 | 2.95E-04 | 4.35E-08 | 7.38E-05 | 9.99E-04 | 1.63E-02 |
| uncultured_bacterium_g_Romboutsia | 6.23E-04 | 4.21E-08 | 7.26E-05 | 4.89E-03 | 4.18E-06 | 7.23E-04 | 9.99E-04 | 1.63E-02 |
| uncultured_bacterium_g_[Eubacterium]_ruminantium_group | 2.01E-03 | 5.94E-06 | 8.62E-04 | 0.00E+00 | 0.00E+00 | 0.00E+00 | 9.99E-04 | 1.63E-02 |
| uncultured_bacterium_g_Marvinbryantia | 4.16E-03 | 6.47E-07 | 2.84E-04 | 2.66E-03 | 9.82E-07 | 3.50E-04 | 3.00E-03 | 3.67E-02 |
| uncultured_bacterium_g_Anaerostipes | 4.69E-04 | 1.58E-06 | 4.45E-04 | 3.02E-03 | 6.26E-06 | 8.84E-04 | 1.30E-02 | 1.27E-01 |
| uncultured_bacterium_g_Ruminococcaceae_UCG-013 | 2.67E-03 | 3.61E-06 | 6.72E-04 | 5.38E-04 | 9.70E-08 | 1.10E-04 | 1.30E-02 | 1.27E-01 |
| uncultured_bacterium_g_Clostridium_sensu_stricto_1 | 9.12E-04 | 5.57E-07 | 2.64E-04 | 1.95E-04 | 6.02E-08 | 8.68E-05 | 1.60E-02 | 1.42E-01 |
| uncultured_bacterium_g_Candidatus_Soleaferrea | 6.26E-05 | 1.07E-09 | 1.16E-05 | 1.20E-04 | 3.41E-09 | 2.07E-05 | 2.00E-02 | 1.63E-01 |
| uncultured_bacterium_g_Ruminococcaceae_UCG-009 | 1.09E-03 | 2.47E-07 | 1.76E-04 | 5.64E-04 | 6.84E-08 | 9.24E-05 | 2.20E-02 | 1.66E-01 |
| uncultured_bacterium_f_Eggerthellaceae | 9.54E-05 | 6.54E-09 | 2.86E-05 | 1.82E-04 | 6.21E-09 | 2.79E-05 | 4.00E-02 | 2.80E-01 |
| uncultured_bacterium_g_Candidatus_Stoquefichus | 1.88E-05 | 1.48E-09 | 1.36E-05 | 8.92E-05 | 8.84E-09 | 3.32E-05 | 4.80E-02 | 3.13E-01 |
| uncultured_bacterium_g_Anaerotruncus | 1.36E-03 | 3.59E-07 | 2.12E-04 | 8.08E-04 | 2.21E-07 | 1.66E-04 | 5.39E-02 | 3.30E-01 |
| uncultured_bacterium_g_Faecalibaculum | 1.74E-02 | 1.52E-04 | 4.36E-03 | 9.09E-03 | 1.76E-05 | 1.48E-03 | 6.49E-02 | 3.64E-01 |
| uncultured_bacterium_g_Ruminiclostridium_6 | 3.82E-04 | 9.90E-08 | 1.11E-04 | 7.82E-04 | 2.50E-07 | 1.77E-04 | 6.69E-02 | 3.64E-01 |
| Burkholderiales_bacterium_YL45 | 3.12E-03 | 3.78E-06 | 6.87E-04 | 1.76E-03 | 7.27E-07 | 3.01E-04 | 8.69E-02 | 4.48E-01 |
| uncultured_bacterium_g_Shuttleworthia | 1.41E-03 | 2.66E-06 | 5.77E-04 | 3.18E-03 | 6.43E-06 | 8.97E-04 | 1.24E-01 | 4.57E-01 |
| uncultured_bacterium_g_Ruminiclostridium_9 | 6.14E-03 | 5.96E-06 | 8.63E-04 | 4.39E-03 | 3.75E-06 | 6.84E-04 | 1.26E-01 | 4.57E-01 |
| uncultured_bacterium_g_Staphylococcus | 6.06E-05 | 1.71E-09 | 1.46E-05 | 4.41E-03 | 1.16E-04 | 3.81E-03 | 1.26E-01 | 4.57E-01 |
| uncultured_bacterium_g_Ruminiclostridium | 5.25E-03 | 1.72E-05 | 1.47E-03 | 2.76E-03 | 1.80E-06 | 4.75E-04 | 1.31E-01 | 4.57E-01 |
| Enterococcus_faecalis | 8.85E-05 | 5.04E-09 | 2.51E-05 | 1.92E-03 | 2.01E-05 | 1.59E-03 | 1.33E-01 | 4.57E-01 |
| uncultured_bacterium_g_Family_XIII_UCG-001 | 1.02E-04 | 5.15E-09 | 2.54E-05 | 1.50E-04 | 1.77E-09 | 1.49E-05 | 1.35E-01 | 4.57E-01 |
| uncultured_bacterium_f_Peptococcaceae | 4.95E-04 | 8.59E-08 | 1.04E-04 | 3.23E-04 | 1.70E-08 | 4.62E-05 | 1.39E-01 | 4.57E-01 |
| uncultured_bacterium_g_Blautia | 2.37E-03 | 2.85E-06 | 5.97E-04 | 1.36E-03 | 6.96E-07 | 2.95E-04 | 1.39E-01 | 4.57E-01 |
| uncultured_bacterium_g_Candidatus_Saccharimonas | 9.98E-03 | 1.34E-05 | 1.29E-03 | 7.21E-03 | 1.32E-05 | 1.28E-03 | 1.47E-01 | 4.57E-01 |
| uncultured_bacterium_g_Lysinibacillus | 4.93E-05 | 1.27E-09 | 1.26E-05 | 9.19E-05 | 4.68E-09 | 2.42E-05 | 1.47E-01 | 4.57E-01 |
| Mucispirillum_schaedleri_ASF457 | 1.51E-03 | 1.11E-06 | 3.72E-04 | 1.42E-02 | 6.65E-04 | 9.12E-03 | 1.55E-01 | 4.57E-01 |
| uncultured_bacterium_g_[Ruminococcus]_gauvreauii_group | 2.00E-04 | 2.58E-09 | 1.80E-05 | 3.60E-04 | 8.13E-08 | 1.01E-04 | 1.56E-01 | 4.57E-01 |
| uncultured_bacterium_g_GCA-900066575 | 3.71E-03 | 2.38E-06 | 5.46E-04 | 2.59E-03 | 2.36E-06 | 5.43E-04 | 1.59E-01 | 4.57E-01 |
| uncultured_bacterium_g_Parvibacter | 7.70E-04 | 1.51E-07 | 1.37E-04 | 5.34E-04 | 5.87E-08 | 8.56E-05 | 1.59E-01 | 4.57E-01 |
| uncultured_bacterium_g_Lachnospiraceae_FCS020_group | 2.00E-03 | 1.24E-06 | 3.94E-04 | 1.38E-03 | 3.92E-07 | 2.21E-04 | 1.63E-01 | 4.57E-01 |
| uncultured_bacterium_g_[Eubacterium]_xylanophilum_group | 3.31E-03 | 4.06E-06 | 7.12E-04 | 2.09E-03 | 1.14E-06 | 3.78E-04 | 1.64E-01 | 4.57E-01 |
| uncultured_bacterium_g_Solibacillus | 2.13E-04 | 1.55E-08 | 4.41E-05 | 2.96E-04 | 1.04E-08 | 3.61E-05 | 1.68E-01 | 4.57E-01 |
| uncultured_bacterium_g_Jeotgalicoccus | 3.27E-06 | 3.69E-11 | 2.15E-06 | 3.13E-05 | 4.79E-09 | 2.45E-05 | 1.82E-01 | 4.82E-01 |
| uncultured_bacterium_f_Muribaculaceae | 2.65E-01 | 1.98E-03 | 1.58E-02 | 2.39E-01 | 9.13E-04 | 1.07E-02 | 2.01E-01 | 5.18E-01 |
| uncultured_bacterium_g_Butyricicoccus | 1.08E-03 | 9.34E-07 | 3.42E-04 | 6.42E-04 | 7.89E-08 | 9.93E-05 | 2.26E-01 | 5.50E-01 |
| uncultured_bacterium_g_Oscillibacter | 4.36E-03 | 9.88E-06 | 1.11E-03 | 2.80E-03 | 2.94E-06 | 6.06E-04 | 2.28E-01 | 5.50E-01 |
| uncultured_bacterium_g_Prevotellaceae_UCG-001 | 1.36E-02 | 7.82E-05 | 3.13E-03 | 9.36E-03 | 2.32E-05 | 1.70E-03 | 2.36E-01 | 5.50E-01 |
| uncultured_bacterium_g_Lachnospiraceae_UCG-006 | 2.44E-02 | 2.50E-04 | 5.59E-03 | 1.52E-02 | 1.44E-04 | 4.24E-03 | 2.43E-01 | 5.50E-01 |
| uncultured_bacterium_f_Erysipelotrichaceae | 6.10E-04 | 1.54E-07 | 1.39E-04 | 4.24E-04 | 3.72E-08 | 6.82E-05 | 2.46E-01 | 5.50E-01 |
| uncultured_bacterium_g_Turicibacter | 2.55E-02 | 2.20E-04 | 5.25E-03 | 1.77E-02 | 1.20E-04 | 3.87E-03 | 2.62E-01 | 5.50E-01 |
| uncultured_bacterium_g_Ruminiclostridium_5 | 2.89E-03 | 5.74E-06 | 8.47E-04 | 1.80E-03 | 7.99E-07 | 3.16E-04 | 2.64E-01 | 5.50E-01 |
| uncultured_bacterium_g_Bacteroides | 5.11E-03 | 8.03E-06 | 1.00E-03 | 3.43E-03 | 1.02E-05 | 1.13E-03 | 2.67E-01 | 5.50E-01 |
| uncultured_bacterium_g_Tyzzerella | 5.81E-04 | 4.22E-07 | 2.30E-04 | 3.17E-04 | 2.57E-08 | 5.67E-05 | 2.69E-01 | 5.50E-01 |
| uncultured_bacterium_g_Muribaculum | 4.85E-03 | 1.95E-06 | 4.93E-04 | 3.63E-03 | 6.60E-06 | 9.08E-04 | 2.72E-01 | 5.50E-01 |
| uncultured_bacterium_g_[Eubacterium]_nodatum_group | 4.52E-04 | 3.87E-08 | 6.95E-05 | 5.57E-04 | 3.27E-08 | 6.39E-05 | 2.77E-01 | 5.50E-01 |
| uncultured_bacterium_g_Family_XIII_AD3011_group | 6.72E-04 | 4.20E-08 | 7.24E-05 | 5.72E-04 | 2.52E-08 | 5.61E-05 | 2.87E-01 | 5.50E-01 |
| uncultured_bacterium_g_Ruminococcaceae_NK4A214_group | 5.63E-04 | 6.31E-08 | 8.88E-05 | 4.39E-04 | 3.33E-08 | 6.45E-05 | 2.88E-01 | 5.50E-01 |
| uncultured_bacterium_f_Christensenellaceae | 3.17E-04 | 1.52E-08 | 4.36E-05 | 2.54E-04 | 9.82E-09 | 3.50E-05 | 2.96E-01 | 5.50E-01 |
| uncultured_bacterium_f_Lachnospiraceae | 5.73E-02 | 9.84E-04 | 1.11E-02 | 4.33E-02 | 3.29E-04 | 6.41E-03 | 2.98E-01 | 5.50E-01 |
| uncultured_bacterium_g_Parasutterella | 1.34E-03 | 6.43E-07 | 2.83E-04 | 3.54E-03 | 3.32E-05 | 2.04E-03 | 3.45E-01 | 6.25E-01 |
| uncultured_bacterium_o_Rhodospirillales | 3.29E-04 | 3.16E-08 | 6.29E-05 | 6.48E-04 | 7.51E-07 | 3.06E-04 | 4.12E-01 | 7.30E-01 |
| uncultured_bacterium_g_[Eubacterium]_coprostanoligenes_group | 5.69E-04 | 1.54E-07 | 1.39E-04 | 4.17E-04 | 1.12E-07 | 1.18E-04 | 4.21E-01 | 7.30E-01 |
| uncultured_bacterium_g_A2 | 2.56E-03 | 1.45E-06 | 4.26E-04 | 2.15E-03 | 7.79E-07 | 3.12E-04 | 4.25E-01 | 7.30E-01 |
| uncultured_bacterium_g_UBA1819 | 2.82E-04 | 1.31E-08 | 4.04E-05 | 3.52E-04 | 5.01E-08 | 7.92E-05 | 4.36E-01 | 7.36E-01 |
| uncultured_bacterium_g_Klebsiella | 3.52E-03 | 7.07E-05 | 2.97E-03 | 1.44E-03 | 1.48E-05 | 1.36E-03 | 4.69E-01 | 7.61E-01 |
| uncultured_bacterium_g_Akkermansia | 1.16E-03 | 4.63E-07 | 2.41E-04 | 1.79E-03 | 3.98E-06 | 7.05E-04 | 4.71E-01 | 7.61E-01 |
| uncultured_bacterium_g_Tyzzerella_3 | 2.55E-04 | 4.47E-08 | 7.48E-05 | 1.88E-04 | 3.46E-08 | 6.58E-05 | 4.81E-01 | 7.61E-01 |
| uncultured_bacterium_g_Acetatifactor | 3.44E-04 | 7.52E-08 | 9.69E-05 | 4.90E-04 | 2.23E-07 | 1.67E-04 | 4.82E-01 | 7.61E-01 |
| uncultured_bacterium_g_GCA-900066225 | 8.55E-05 | 4.12E-09 | 2.27E-05 | 6.71E-05 | 2.93E-09 | 1.91E-05 | 5.09E-01 | 7.90E-01 |
| Acinetobacter_johnsonii | 4.91E-05 | 2.61E-09 | 1.81E-05 | 4.20E-04 | 1.11E-06 | 3.73E-04 | 5.22E-01 | 7.90E-01 |
| uncultured_bacterium_g_Alistipes | 7.13E-03 | 4.13E-05 | 2.27E-03 | 5.11E-03 | 9.24E-06 | 1.07E-03 | 5.30E-01 | 7.90E-01 |
| uncultured_bacterium_f_Desulfovibrionaceae | 3.24E-02 | 1.56E-04 | 4.42E-03 | 2.66E-02 | 4.77E-04 | 7.72E-03 | 5.35E-01 | 7.90E-01 |
| uncultured_bacterium_g_Lachnospiraceae_UCG-001 | 3.34E-03 | 4.45E-06 | 7.46E-04 | 2.54E-03 | 7.67E-06 | 9.79E-04 | 5.41E-01 | 7.90E-01 |
| Monocercomonoides_sp._PA203 | 5.55E-04 | 6.45E-08 | 8.98E-05 | 4.46E-04 | 1.89E-07 | 1.54E-04 | 5.56E-01 | 7.90E-01 |
| uncultured_bacterium_g_Odoribacter | 8.80E-04 | 6.42E-07 | 2.83E-04 | 6.32E-04 | 1.05E-07 | 1.14E-04 | 5.59E-01 | 7.90E-01 |
| uncultured_bacterium_g_Intestinimonas | 2.63E-04 | 1.95E-08 | 4.94E-05 | 2.23E-04 | 2.29E-08 | 5.36E-05 | 5.72E-01 | 7.90E-01 |
| uncultured_bacterium_g_Roseburia | 2.14E-02 | 1.39E-04 | 4.17E-03 | 2.79E-02 | 4.67E-04 | 7.64E-03 | 5.72E-01 | 7.90E-01 |
| uncultured_bacterium_o_Gastranaerophilales | 1.25E-03 | 4.62E-07 | 2.40E-04 | 2.70E-03 | 1.98E-05 | 1.57E-03 | 6.11E-01 | 8.27E-01 |
| uncultured_bacterium_g_Bilophila | 1.46E-04 | 5.21E-09 | 2.55E-05 | 1.29E-04 | 4.21E-09 | 2.29E-05 | 6.25E-01 | 8.27E-01 |
| uncultured_bacterium_g_Lachnospiraceae_UCG-008 | 1.23E-03 | 6.89E-07 | 2.93E-04 | 1.44E-03 | 9.78E-07 | 3.50E-04 | 6.42E-01 | 8.27E-01 |
| uncultured_bacterium_g_Erysipelatoclostridium | 1.08E-03 | 5.34E-07 | 2.58E-04 | 1.25E-03 | 5.01E-07 | 2.50E-04 | 6.49E-01 | 8.27E-01 |
| uncultured_bacterium_g_ASF356 | 2.16E-03 | 6.68E-06 | 9.14E-04 | 1.78E-03 | 4.25E-06 | 7.29E-04 | 6.56E-01 | 8.27E-01 |
| uncultured_bacterium_g_Dubosiella | 3.47E-02 | 6.56E-04 | 9.05E-03 | 4.02E-02 | 7.00E-04 | 9.35E-03 | 6.56E-01 | 8.27E-01 |
| uncultured_bacterium_g_Coriobacteriaceae_UCG-002 | 1.81E-03 | 2.75E-06 | 5.86E-04 | 1.54E-03 | 5.40E-07 | 2.60E-04 | 6.61E-01 | 8.27E-01 |
| uncultured_bacterium_g_Negativibacillus | 2.01E-04 | 9.63E-09 | 3.47E-05 | 1.80E-04 | 8.34E-09 | 3.23E-05 | 6.66E-01 | 8.27E-01 |
| uncultured_bacterium_g_Ruminococcaceae_UCG-010 | 1.03E-03 | 4.64E-08 | 7.62E-05 | 1.14E-03 | 3.64E-07 | 2.13E-04 | 6.84E-01 | 8.32E-01 |
| uncultured_bacterium_g_Escherichia-Shigella | 1.08E-02 | 5.55E-04 | 8.33E-03 | 2.75E-02 | 4.91E-03 | 2.48E-02 | 7.01E-01 | 8.32E-01 |
| uncultured_bacterium_f_Clostridiales_vadinBB60_group | 3.16E-04 | 1.54E-07 | 1.39E-04 | 2.08E-04 | 7.22E-09 | 3.00E-05 | 7.02E-01 | 8.32E-01 |
| uncultured_bacterium_f_Ruminococcaceae | 7.49E-03 | 8.47E-06 | 1.03E-03 | 8.00E-03 | 1.02E-05 | 1.13E-03 | 7.04E-01 | 8.32E-01 |
| uncultured_bacterium_g_Lactobacillus | 1.59E-01 | 5.47E-03 | 2.61E-02 | 1.45E-01 | 5.27E-03 | 2.57E-02 | 7.18E-01 | 8.38E-01 |
| uncultured_bacterium_g_Alloprevotella | 2.40E-02 | 2.07E-04 | 5.08E-03 | 2.17E-02 | 3.04E-04 | 6.16E-03 | 7.60E-01 | 8.77E-01 |
| uncultured_bacterium_o_Mollicutes_RF39 | 1.15E-03 | 1.92E-06 | 4.89E-04 | 8.71E-04 | 9.11E-08 | 1.07E-04 | 8.73E-01 | 9.66E-01 |
| uncultured_bacterium_g_[Eubacterium]_hallii_group | 1.03E-04 | 7.77E-09 | 3.12E-05 | 1.09E-04 | 6.71E-09 | 2.90E-05 | 8.76E-01 | 9.66E-01 |
| uncultured_bacterium_g_Ruminococcus_1 | 3.56E-03 | 2.40E-06 | 5.48E-04 | 3.36E-03 | 9.96E-06 | 1.12E-03 | 8.83E-01 | 9.66E-01 |
| uncultured_bacterium_g_Lachnoclostridium | 4.83E-03 | 1.88E-05 | 1.53E-03 | 4.54E-03 | 4.55E-06 | 7.54E-04 | 9.11E-01 | 9.66E-01 |
| uncultured_bacterium_f_Blattella_germanica_German_cockroach | 5.14E-05 | 2.90E-09 | 1.91E-05 | 4.82E-05 | 5.09E-09 | 2.52E-05 | 9.16E-01 | 9.66E-01 |
| uncultured_bacterium_g_Ruminococcaceae_UCG-014 | 1.12E-01 | 8.05E-03 | 3.17E-02 | 1.16E-01 | 2.98E-03 | 1.93E-02 | 9.27E-01 | 9.66E-01 |
| uncultured_bacterium_g_Desulfovibrio | 1.30E-03 | 1.04E-06 | 3.60E-04 | 1.25E-03 | 1.34E-06 | 4.09E-04 | 9.29E-01 | 9.66E-01 |
| uncultured_bacterium_g_Lachnospiraceae_NK4A136_group | 6.66E-02 | 7.24E-04 | 9.52E-03 | 6.81E-02 | 1.41E-03 | 1.33E-02 | 9.30E-01 | 9.66E-01 |
| uncultured_bacterium_g_Rikenellaceae_RC9_gut_group | 2.99E-03 | 2.16E-06 | 5.19E-04 | 3.09E-03 | 9.70E-06 | 1.10E-03 | 9.45E-01 | 9.66E-01 |
| uncultured_bacterium_g_Enterorhabdus | 1.33E-03 | 5.95E-07 | 2.73E-04 | 1.32E-03 | 1.34E-07 | 1.29E-04 | 9.50E-01 | 9.66E-01 |
| uncultured_bacterium_g_[Eubacterium]_brachy_group | 2.41E-04 | 5.38E-08 | 8.20E-05 | 2.35E-04 | 1.91E-08 | 4.88E-05 | 9.52E-01 | 9.66E-01 |
| uncultured_bacterium_g_Defluviitaleaceae_UCG-011 | 1.91E-03 | 2.86E-07 | 1.89E-04 | 1.89E-03 | 7.06E-07 | 2.97E-04 | 9.56E-01 | 9.66E-01 |
| uncultured_bacterium_g_Gordonibacter | 1.61E-04 | 1.09E-08 | 3.69E-05 | 1.61E-04 | 3.26E-09 | 2.02E-05 | 9.97E-01 | 9.97E-01 |
