# Supplementary material for: Fisetin Regulates Gut Microbiota and Exerts Neuroprotective Effect on Mouse Model of Parkinson’s Disease
Source: Front Neurosci. 2020 Dec 14;14:549037. doi: 10.3389/fnins.2020.549037 (PMC7768012; doi:10.3389/fnins.2020.549037)
Supplement: Supplementary file 3 [file Table_2.docx]

Supplementary Material

# Supplementary Tables

## Supplementary table S2: KEGG functional prediction analysis (F vs MPTP)

| Class1 | Class2 | MPTP1 | F1 | MPTP1: parent seq. count | F1: parent seq. count | MPTP1: rel. freq. (%) | F1: rel. freq. (%) | p-values | p-values (corrected) | Effect size | 95.0% lower CI | 95.0% upper CI |
| --- | --- | --- | --- | --- | --- | --- | --- | --- | --- | --- | --- | --- |
| Metabolism | Carbohydrate metabolism | 5608605 | 6070313 | 37156904 | 37362192 | 15.09438 | 16.24721 | 0 | 0 | -1.15283 | -1.16934 | -1.13632 |
| Metabolism | Lipid metabolism | 1257630 | 1290860 | 37156904 | 37362192 | 3.384647 | 3.45499 | 0 | 0 | -0.07034 | -0.0786 | -0.06209 |
| Metabolism | Metabolism of cofactors and vitamins | 2360228 | 2381615 | 37156904 | 37362192 | 6.352058 | 6.374398 | 7.80E-05 | 9.87E-05 | -0.02234 | -0.03343 | -0.01125 |
| Metabolism | Energy metabolism | 2611704 | 2458921 | 37156904 | 37362192 | 7.028853 | 6.581308 | 0 | 0 | 0.447544 | 0.436103 | 0.458985 |
| Metabolism | Nucleotide metabolism | 2266848 | 2073957 | 37156904 | 37362192 | 6.100745 | 5.550951 | 0 | 0 | 0.549794 | 0.539153 | 0.560436 |
| Metabolism | Biosynthesis of other secondary metabolites | 449486 | 507362 | 37156904 | 37362192 | 1.209697 | 1.357956 | 0 | 0 | -0.14826 | -0.15338 | -0.14314 |
| Metabolism | Amino acid metabolism | 3957831 | 4099807 | 37156904 | 37362192 | 10.65167 | 10.97314 | 0 | 0 | -0.32147 | -0.33558 | -0.30737 |
| Metabolism | Metabolism of terpenoids and polyketides | 716609 | 671513 | 37156904 | 37362192 | 1.928603 | 1.797306 | 0 | 0 | 0.131296 | 0.125151 | 0.137442 |
| Metabolism | Xenobiotics biodegradation and metabolism | 685722 | 786893 | 37156904 | 37362192 | 1.845477 | 2.106121 | 0 | 0 | -0.26064 | -0.26697 | -0.25432 |
| Metabolism | Metabolism of other amino acids | 759146 | 860910 | 37156904 | 37362192 | 2.043082 | 2.304228 | 0 | 0 | -0.26115 | -0.26777 | -0.25452 |
| Metabolism | Glycan biosynthesis and metabolism | 883233 | 990464 | 37156904 | 37362192 | 2.377036 | 2.650979 | 0 | 0 | -0.27394 | -0.28106 | -0.26683 |
| Genetic Information Processing | Translation | 2186086 | 1819355 | 37156904 | 37362192 | 5.883391 | 4.869508 | 0 | 0 | 1.013883 | 1.003637 | 1.024129 |
| Metabolism | Global and overview maps | 5180598 | 5006498 | 37156904 | 37362192 | 13.94249 | 13.3999 | 0 | 0 | 0.542587 | 0.526981 | 0.558192 |
| Human Diseases | Drug resistance | 290578 | 283729 | 37156904 | 37362192 | 0.78203 | 0.759401 | 0 | 0 | 0.022628 | 0.018652 | 0.026605 |
| Environmental Information Processing | Membrane transport | 1801953 | 2110596 | 37156904 | 37362192 | 4.849578 | 5.649015 | 0 | 0 | -0.79944 | -0.80957 | -0.78931 |
| Environmental Information Processing | Signal transduction | 1097399 | 1237926 | 37156904 | 37362192 | 2.953419 | 3.313312 | 0 | 0 | -0.35989 | -0.36781 | -0.35198 |
| Cellular Processes | Cell motility | 578821 | 591818 | 37156904 | 37362192 | 1.557775 | 1.584002 | 0 | 0 | -0.02623 | -0.03188 | -0.02058 |
| Genetic Information Processing | Folding, sorting and degradation | 816301 | 744603 | 37156904 | 37362192 | 2.196903 | 1.992932 | 0 | 0 | 0.203971 | 0.197462 | 0.21048 |
| Genetic Information Processing | Transcription | 99603 | 76669 | 37156904 | 37362192 | 0.268061 | 0.205205 | 0 | 0 | 0.062856 | 0.060644 | 0.065068 |
| Genetic Information Processing | Replication and repair | 1846207 | 1597151 | 37156904 | 37362192 | 4.968678 | 4.274779 | 0 | 0 | 0.6939 | 0.684361 | 0.703438 |
| Organismal Systems | Endocrine system | 183941 | 173621 | 37156904 | 37362192 | 0.495039 | 0.464697 | 0 | 0 | 0.030342 | 0.027198 | 0.033485 |
| Environmental Information Processing | Signaling molecules and interaction | 0 | 0 | 37156904 | 37362192 | 0 | 0 | 1 | 1.075 | 0 | ####### | 5.26E-06 |
| Cellular Processes | Cell growth and death | 302838 | 252793 | 37156904 | 37362192 | 0.815025 | 0.676601 | 0 | 0 | 0.138424 | 0.134511 | 0.142336 |
| Cellular Processes | Transport and catabolism | 161505 | 203561 | 37156904 | 37362192 | 0.434657 | 0.544832 | 0 | 0 | -0.11017 | -0.11335 | -0.107 |
| Organismal Systems | Circulatory system | 42 | 84 | 37156904 | 37362192 | 0.000113 | 0.000225 | 0.000179 | 0.00022 | -0.00011 | -0.00018 | ####### |
| Organismal Systems | Development | 0 | 0 | 37156904 | 37362192 | 0 | 0 | 1 | 1.04878 | 0 | ####### | 5.26E-06 |
| Cellular Processes | Cellular community | 0 | 0 | 37156904 | 37362192 | 0 | 0 | 1 | 1.02381 | 0 | ####### | 5.26E-06 |
| Organismal Systems | Immune system | 45246 | 44256 | 37156904 | 37362192 | 0.12177 | 0.118451 | 3.54E-05 | 4.75E-05 | 0.003319 | 0.001741 | 0.004897 |
| Organismal Systems | Environmental adaptation | 71629 | 69493 | 37156904 | 37362192 | 0.192774 | 0.185998 | 1.73E-11 | 2.40E-11 | 0.006776 | 0.004797 | 0.008756 |
| Organismal Systems | Nervous system | 108466 | 110732 | 37156904 | 37362192 | 0.291913 | 0.296374 | 0.000377 | 0.000451 | -0.00446 | -0.00693 | -0.002 |
| Organismal Systems | Sensory system | 0 | 0 | 37156904 | 37362192 | 0 | 0 | 1 | 1 | 0 | ####### | 5.26E-06 |
| Human Diseases | Endocrine and metabolic diseases | 61850 | 55028 | 37156904 | 37362192 | 0.166456 | 0.147283 | 0 | 0 | 0.019174 | 0.017371 | 0.020976 |
| Organismal Systems | Excretory system | 8031 | 15325 | 37156904 | 37362192 | 0.021614 | 0.041017 | 0 | 0 | -0.0194 | -0.02021 | -0.0186 |
| Organismal Systems | Digestive system | 15439 | 15307 | 37156904 | 37362192 | 0.041551 | 0.040969 | 0.21642 | 0.238616 | 0.000582 | -0.00035 | 0.001509 |
| Human Diseases | Neurodegenerative diseases | 65860 | 83788 | 37156904 | 37362192 | 0.177248 | 0.224259 | 0 | 0 | -0.04701 | -0.04905 | -0.04497 |
| Human Diseases | Substance dependence | 3915 | 4950 | 37156904 | 37362192 | 0.010536 | 0.013249 | 0 | 0 | -0.00271 | -0.00321 | -0.00221 |
| Human Diseases | Infectious diseases: Bacterial | 312262 | 308296 | 37156904 | 37362192 | 0.840388 | 0.825155 | 4.66E-13 | 6.91E-13 | 0.015233 | 0.011101 | 0.019364 |
| Human Diseases | Infectious diseases: Parasitic | 8223 | 8701 | 37156904 | 37362192 | 0.02213 | 0.023288 | 0.000911 | 0.001059 | -0.00116 | -0.00185 | -0.00047 |
| Human Diseases | Infectious diseases: Viral | 461 | 272 | 37156904 | 37362192 | 0.001241 | 0.000728 | 1.30E-12 | 1.86E-12 | 0.000513 | 0.000365 | 0.00066 |
| Human Diseases | Cancers: Overview | 302861 | 301456 | 37156904 | 37362192 | 0.815087 | 0.806848 | 7.34E-05 | 9.56E-05 | 0.008239 | 0.004161 | 0.012317 |
| Human Diseases | Cancers: Specific types | 27781 | 29771 | 37156904 | 37362192 | 0.074767 | 0.079682 | 2.21E-14 | 3.39E-14 | -0.00492 | -0.00618 | -0.00365 |
| Human Diseases | Immune diseases | 21955 | 23770 | 37156904 | 37362192 | 0.059087 | 0.06362 | 2.78E-15 | 4.42E-15 | -0.00453 | -0.00566 | -0.0034 |
| Human Diseases | Cardiovascular diseases | 11 | 28 | 37156904 | 37362192 | 2.96E-05 | 7.49E-05 | 0.009486 | 0.010734 | ####### | ####### | ####### |
