# Supplementary material for: Fisetin Regulates Gut Microbiota and Exerts Neuroprotective Effect on Mouse Model of Parkinson’s Disease
Source: Front Neurosci. 2020 Dec 14;14:549037. doi: 10.3389/fnins.2020.549037 (PMC7768012; doi:10.3389/fnins.2020.549037)
Supplement: Supplementary file 4 [file Table_3.docx]

Supplementary Material

# Supplementary Tables

## Supplementary table S3: Clusters of orthologous groups (COGs) of proteins functional prediction analysis (F vs MPTP)

| Class1 | Class2 | MPTP1 | F1 | MPTP1: parent seq. count | F1: parent seq. count | MPTP1: rel. freq. (%) | F1: rel. freq. (%) | p-values | p-values (corrected) | Effect size | 95.0% lower CI | 95.0% upper CI |
| --- | --- | --- | --- | --- | --- | --- | --- | --- | --- | --- | --- | --- |
| INFORMATION STORAGE AND PROCESSING | RNA processing and modification | 3239 | 6763 | 53789229 | 57713501 | 0.006022 | 0.011718 | 0 | 0 | -0.0057 | -0.00605 | -0.00535 |
| INFORMATION STORAGE AND PROCESSING | Chromatin structure and dynamics | 6425 | 3803 | 53789229 | 57713501 | 0.011945 | 0.006589 | 0 | 0 | 0.005355 | 0.004992 | 0.005718 |
| METABOLISM | Energy production and conversion | 3007439 | 3154967 | 53789229 | 57713501 | 5.591155 | 5.466601 | 0 | 0 | 0.124553 | 0.116059 | 0.133048 |
| CELLULAR PROCESSES AND SIGNALING | Cell cycle control, cell division, chromosome partitioning | 739349 | 709848 | 53789229 | 57713501 | 1.37453 | 1.229951 | 0 | 0 | 0.144578 | 0.14036 | 0.148797 |
| METABOLISM | Amino acid transport and metabolism | 4268596 | 4525244 | 53789229 | 57713501 | 7.935782 | 7.840876 | 0 | 0 | 0.094906 | 0.084889 | 0.104923 |
| METABOLISM | Nucleotide transport and metabolism | 1709355 | 1597284 | 53789229 | 57713501 | 3.177876 | 2.767609 | 0 | 0 | 0.410267 | 0.403948 | 0.416586 |
| METABOLISM | Carbohydrate transport and metabolism | 4372101 | 5826840 | 53789229 | 57713501 | 8.128209 | 10.09615 | 0 | 0 | -1.96794 | -1.97861 | -1.95727 |
| METABOLISM | Coenzyme transport and metabolism | 2176311 | 2275953 | 53789229 | 57713501 | 4.045998 | 3.943537 | 0 | 0 | 0.102461 | 0.095182 | 0.109741 |
| METABOLISM | Lipid transport and metabolism | 1297672 | 1328730 | 53789229 | 57713501 | 2.412513 | 2.302286 | 0 | 0 | 0.110226 | 0.104585 | 0.115868 |
| INFORMATION STORAGE AND PROCESSING | Translation, ribosomal structure and biogenesis | 3847843 | 3436048 | 53789229 | 57713501 | 7.153557 | 5.953629 | 0 | 0 | 1.199927 | 1.19072 | 1.209134 |
| INFORMATION STORAGE AND PROCESSING | Transcription | 4255842 | 4928451 | 53789229 | 57713501 | 7.912071 | 8.539511 | 0 | 0 | -0.62744 | -0.63764 | -0.61724 |
| INFORMATION STORAGE AND PROCESSING | Replication, recombination and repair | 3776586 | 3556779 | 53789229 | 57713501 | 7.021082 | 6.16282 | 0 | 0 | 0.858263 | 0.849033 | 0.867492 |
| CELLULAR PROCESSES AND SIGNALING | Cell wall/membrane/envelope biogenesis | 3584219 | 3784505 | 53789229 | 57713501 | 6.663451 | 6.5574 | 0 | 0 | 0.106051 | 0.096817 | 0.115285 |
| CELLULAR PROCESSES AND SIGNALING | Cell motility | 794236 | 845319 | 53789229 | 57713501 | 1.476571 | 1.464682 | 1.87E-07 | 1.95E-07 | 0.011889 | 0.007414 | 0.016364 |
| CELLULAR PROCESSES AND SIGNALING | Posttranslational modification, protein turnover, chaperones | 1732419 | 1781832 | 53789229 | 57713501 | 3.220754 | 3.087375 | 0 | 0 | 0.13338 | 0.126882 | 0.139878 |
| METABOLISM | Inorganic ion transport and metabolism | 2338828 | 2725674 | 53789229 | 57713501 | 4.348134 | 4.722767 | 0 | 0 | -0.37463 | -0.38236 | -0.36691 |
| METABOLISM | Secondary metabolites biosynthesis, transport and catabolism | 489475 | 669067 | 53789229 | 57713501 | 0.909987 | 1.15929 | 0 | 0 | -0.2493 | -0.25306 | -0.24555 |
| POORLY CHARACTERIZED | General function prediction only | 6117831 | 6442428 | 53789229 | 57713501 | 11.37371 | 11.16277 | 0 | 0 | 0.210936 | 0.199185 | 0.222686 |
| POORLY CHARACTERIZED | Function unknown | 3636159 | 4013232 | 53789229 | 57713501 | 6.760013 | 6.953714 | 0 | 0 | -0.1937 | -0.20309 | -0.18431 |
| CELLULAR PROCESSES AND SIGNALING | Signal transduction mechanisms | 3077709 | 3273879 | 53789229 | 57713501 | 5.721794 | 5.67264 | 0 | 0 | 0.049154 | 0.04054 | 0.057769 |
| CELLULAR PROCESSES AND SIGNALING | Intracellular trafficking, secretion, and vesicular transport | 1063934 | 1209595 | 53789229 | 57713501 | 1.977968 | 2.095861 | 0 | 0 | -0.11789 | -0.12314 | -0.11264 |
| CELLULAR PROCESSES AND SIGNALING | Defense mechanisms | 1491544 | 1610308 | 53789229 | 57713501 | 2.772942 | 2.790176 | 3.21E-08 | 3.49E-08 | -0.01723 | -0.02335 | -0.01112 |
| CELLULAR PROCESSES AND SIGNALING | Extracellular structures | 4 | 641 | 53789229 | 57713501 | 7.44E-06 | 0.001111 | ####### | ####### | -0.0011 | -0.00119 | -0.00101 |
| CELLULAR PROCESSES AND SIGNALING | Nuclear structure | 0 | 0 | 53789229 | 57713501 | 0 | 0 | 1 | 1 | 0 | ####### | 3.52E-06 |
| CELLULAR PROCESSES AND SIGNALING | Cytoskeleton | 2113 | 6311 | 53789229 | 57713501 | 0.003928 | 0.010935 | 0 | 0 | -0.00701 | -0.00733 | -0.00669 |
